# Insect Cuticular Hydrocarbons as Dynamic Traits in Sexual Communication

**DOI:** 10.3390/insects6030732

**Published:** 2015-08-04

**Authors:** Fiona C. Ingleby

**Affiliations:** School of Life Sciences, University of Sussex, John Maynard Smith Building, Falmer, Brighton BN1 9QG, UK; E-Mail: f.ingleby@sussex.ac.uk

**Keywords:** CHCs, plasticity, sexual signals, social environment

## Abstract

Recent research has demonstrated extensive within-species variation in pheromone expression in insect species, contrary to the view that pheromones are largely invariant within species. In fact, many studies on insect cuticular hydrocarbons (CHCs) show that pheromones can be highly dynamic traits that can express significant short-term plasticity across both abiotic and social environments. It is likely that this variability in CHC expression contributes to their important role in sexual signaling and mate choice. In this review, I discuss CHC plasticity and how this might influence sexual communication. I also highlight two important avenues for future research: examining plasticity in how individuals respond to CHC signals, and testing how sexual communication varies across abiotic and social environments.

## 1. Introduction

**Figure 1:**
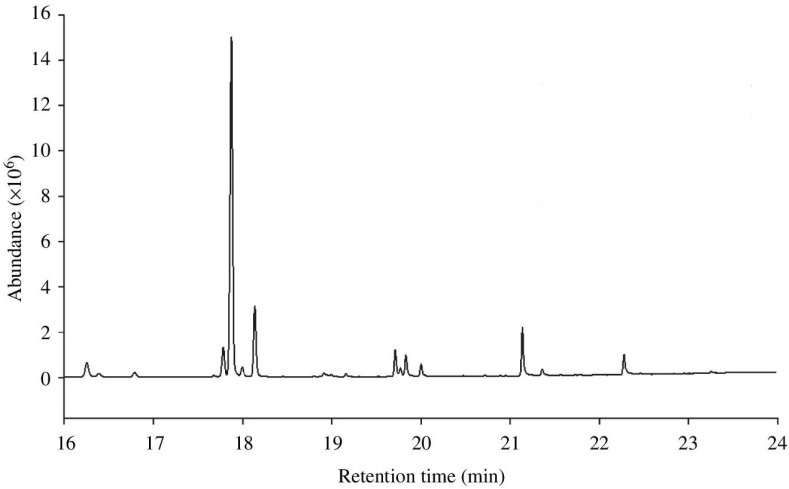
Example of a typical gas chromatograph output from a sample of *Drosophila simulans* CHCs. Each peak represents a different CHC component of the CHC profile, and the integrated peak area gives the abundance of that CHC in the sample. Smaller, relatively volatile CHCs have a lower retention time, and therefore appear first on the chromatograph, as they vaporize and are separated more quickly than larger, more stable CHC molecules.

Across many insect species, cuticular hydrocarbons (CHCs) have a fundamental protective role and contribute to resistance to desiccation [1,2]. CHCs are hydrocarbon molecules derived from fatty-acid compounds that are produced on the adult cuticle shortly following eclosion [3], although sub-adult stages are also known to produce some CHCs e.g., [4]. Insects tend to produce a wide range of different hydrocarbons, which together make up the CHC profile. The profile of an insect can be quantified using gas chromatography to separate the different CHCs and measure the quantity of each type of CHC in a sample (see Figure 1). Analysis of CHCs can vary for details see [5,6], but generally CHC data is now high-throughput and tractable, which has doubtlessly contributed to a spate of research in the past decade.

In addition to a protective role on the insect cuticle, CHCs have been identified as important pheromones [7]. A large body of research has examined the role of the insect CHC profile to allow species recognition, enabling insects to identify conspecifics as potential mates (summarized in [4]). As such, CHCs were considered largely invariant within a species, in keeping with the idea that these chemicals act as a species marker. Selection for species recognition works against individuals with profiles too similar to closely-related sympatric species, or, conversely, too dissimilar to their own species (possibly creating balancing selection on species marker traits). For example, reproductive isolation between subspecies of grasshoppers in a hybrid zone was associated with differentiation of CHC profiles [8]; CHC profiles in eight closely-related species of *Drosophila* fruit fly were found to differ significantly between species [9], as were components of the CHC profile in two species of parasitoid wasp, which differed genetically as well as phenotypically [10]; and CHCs were implicated to act in mate recognition between two sympatric sister species of *Drosophila* [11]. More explicitly, Higgie *et al.* [12] used experimental evolution to demonstrate extremely rapid evolution of CHCs in two closely-related sympatric species of *Drosophila* through natural selection for species recognition.

More recently, research has shifted emphasis onto the idea of CHCs as highly multifunctional pheromones, with diverse roles in intraspecific social interactions e.g., [13,14,15] as suggested in some earlier CHC research [16,17,18]. As a result, it is increasingly clear that CHCs are far from being invariant within a species, and are subject to many different modes of selection. First, stemming from the fundamental protective role of CHCs on the insect cuticle, natural selection is thought to favour the production of desiccation resistant compounds (*i.e.*, long-chained, non-volatile CHCs that prevent water loss [19,20]). It has also been suggested that CHCs might also help to protect the insect from infections through the cuticle [21]. As such, CHCs should vary hugely across environments within a species, depending on abiotic conditions and potentially the presence or absence of infectious agents. Second, through a variety of roles in sexual communication within a species, sexual selection on CHCs can be strong, with both males e.g., [22,23,24,25] and females e.g., [26,27,28] known to exhibit mate preference for particular CHC profiles. Different component molecules of CHC profiles have been identified as various types of sexual signals, including signals of mate quality and attractiveness e.g., [29,30], signalling between male competitors e.g., [31,32], and signalling mating status, breeding status and receptivity to mating e.g., [7,33,34]. In addition, CHCs are known to be transferred between males and females during mating interactions [35]. Furthermore, studies have begun to recognize the consequences of selection (both natural and sexual) differing between males and females, and the implications of this for sexual dimorphism of CHC profiles [14,36,37]. In this review, I summarize recent research examining within-species CHC variation. I consider plasticity across both abiotic and social environmental gradients, as well as the potential for complex interactions and trade-offs between the various forms of selection, and my discussion focuses on the implications of within-species CHC plasticity for sexual signalling in insects.

## 2. Variation in CHCs across Abiotic Environments

### 2.1. CHCs as Condition-Dependent Sexual Signals

A multitude of research has considered plasticity of sexual signals as condition-dependent, wherein signal expression provides information about mate quality and the signal acts as an honest indicator of condition see [38,39]. There is significant evidence that CHCs function this way in insects: for example, it is known that CHCs can be costly to produce and, therefore, that the quantity and type of CHCs produced is likely to vary with individual condition [1,40]. In addition, some hydrocarbons obtained through diet appear to be directly incorporated into the CHC profile, as demonstrated, for example, in *D. melanogaster* [3]. Experimental dietary manipulations have shown that CHC profiles vary with diet quality: *D. serrata* males produced different types and quantities of CHCs when adults were provided with and without yeast [41] or with diets of different quality [42], *Gryllodes sigillatus* male CHCs were altered by diet quality [43], and both male and female CHC profiles were altered by raising *D. simulans* larvae on different food types [44]. In addition, *D. mojavensis* CHCs varied when reared on different host plants, and the influence of diet in this case was also shown to vary with age [45]. Condition-dependence of CHCs has also been observed in *D. bunnanda* [46], wasps [47], and ants [48,49].

In terms of how the condition-dependent expression of CHCs might affect mate choice and attractiveness, there are only a few studies that offer insight. Male *D. simulans* reared on different types of diet showed differences in some components of their CHC profiles, and these differences were mirrored by differences in male attractiveness to females [14]. It appears that males were able to produce a more attractive blend of CHCs on one diet than on the other, consistent with condition-dependent sexual signalling. On the other hand, although diet changed the female CHC profile in *D. melanogaster,* female attractiveness to males was not affected [50]. As is too often the case in sexual selection research, the role of the receiver or responder to a sexual signal is a relatively neglected subject, and our understanding of sexual communication focuses too much on only the signaller’s role in a mating interaction (e.g., [14,25]). More studies need to measure the outcome of mate choice in addition to measuring CHC profile, in order to link signal expression with the information contained within the signal, and the effect it has on the receiver’s behaviour [28,29,37].

### 2.2. Potential Trade-Offs between Signalling CHCs and Waterproofing CHCs

A major function of the insect CHC profile is to waterproof the cuticle and prevent desiccation. For this purpose, it is generally beneficial to have a CHC blend rich in long-chained or branched CHCs, which have effective waterproofing properties e.g., [19,20]. The potential, therefore, exists for a trade-off between two opposing sources of selection on CHCs: natural selection for desiccation resistance *versus* sexual selection for CHC attractiveness. Indeed, some studies have found a distinctive pattern of disruptive selection on the balance of short- and long-chained CHCs, which suggests a trade-off between these components of the insect CHC profile e.g., [14,51]. These studies suggest that the most attractive CHC profiles are those with a high content of relatively short-chained CHCs, which may be more volatile or at least more readily transferred between individuals as contact pheromones.

More directly, studies have shown that temperature and humidity influence the types of CHCs produced. Often, higher temperatures and lower humidity are associated with higher investment in long-chained or branched CHCs, as expected due to these heavier compounds being more stable and creating a more effective waterproofing layer. For example, CHC profiles of *D. serrata* and *D. melanogaster* were found to vary along temperature and humidity clines in Australia [52], experimental temperature manipulation demonstrated that male and female CHC profiles varied with temperature in both *D. melanogaster* [53] and *D. simulans* [44], and altering humidity changed male and female *D. melanogaster* CHC expression [20].

### 2.3. The Consequences of Abiotic Environmental Variation in CHCs for Sexual Signalling

In terms of sexual signalling, what are the consequences of abiotic environmental variation in CHC expression? While a definite answer to this question needs clarification through further research and increased focus on the receiver’s behaviour in the signalling interaction, it is likely that abiotic environmental conditions could strongly influence both the strength of sexual selection on signals, and the inherent reliability of the signals to communicate information about their bearer.

If, as evidence in the previous section suggests, CHCs can function as costly condition-dependent sexual signals and their expression depends heavily on abiotic environmental factors (e.g., [41,43]), it is likely that the intensity of sexual selection will vary across environments. Good quality individuals should be more able to bear costly signal traits than their poorer quality competitors, so CHCs could be used as indicators of mate quality in some environments. However, using such indicators could be difficult in either particularly harsh environments, where no individuals at all are able to bear costly traits, or conversely in low-stress environments, where the differences between individuals might be softened, for example, by a plentiful supply of resources [54,55]. Therefore, as a costly sexual signalling trait, CHCs could be subject to weaker sexual selection in either especially harsh or especially low-stress environments. Such patterns of selection are also likely to contribute to the maintenance or depletion of intraspecific genetic variation for CHC signal traits in different environments [55]. These cross-environment dynamics are likely to have more impact on sexual selection and the reliability of sexual signals when genotypes change the ranked order of attractiveness across environments (*i.e.*, a genotype-by-environment interaction with ecological crossover of reaction norms, see [56]).

In this way, there is potential for abiotic factors to interact with sexual selection [57]: either by reinforcing one another, where a condition-dependent trait acts as an honest indicator of fitness and is favoured by both natural and sexual selection, or by opposing one another, where an attractive CHC profile might reduce individual survival. For example, in *D. serrata*, natural and sexual selection reinforced each other on male CHC profiles but worked in opposite directions on female CHCs [40], and in *D. simulans*, selection across different diets seemed to work against sexual selection on male CHCs, but the same was not found across temperature treatments [14]. Clearly, these interactions can be complex.

On the other hand, if CHCs act in a purely Fisherian manner, where sexual signals and preference for those signals coevolve and are enforced by a genetic covariance between signal and preference [58], then plasticity of signal expression across environments has potential to cause signal unreliability. When the environment changes, or individuals move from one environment to another, there is the potential for signals produced under one set of conditions to be received under another, such that the ranked order of individuals in terms of mate quality does not match the ranked order in terms of signal attractiveness [56]. Therefore, an unattractive mate could appear more attractive (and vice versa) if a signal is produced in different environmental conditions from where it is received. This scenario is in fact possible both with Fisherian sexual signals and condition-dependent signals, and the immediate outcome in both cases is unreliable signalling, unless signal plasticity across environments is directly mirrored by response plasticity. In other words, for environment-dependent sexual signals to remain informative and reliable across short-term environmental variation, the response to these signals (often mate preference) must also be environment-dependent, and somehow “track” the expression of the signal across environments [56,59,60].

Few studies have attempted to test the reliability of CHCs as sexual signals across environments. In crickets, male CHCs expressed over different dietary environments reliably signalled mate quality, whereas female CHCs reliably signalled genetic identity [43]. In *D. simulans*, male CHC profiles varied over diet and temperature conditions but attractiveness was still reliably communicated [61]. Sexual selection on CHCs can be strong e.g., [23,24,27], but clearly research will need to directly test the potential consequences of environment-dependent sexual signalling in order to fully understand how environment-dependent CHC signals function and evolve.

Furthermore, although evidence for environment-dependent mate choice in general is widespread [39,62], few studies have integrated CHC signal expression with the receiver’s response over different environments. The studies which do begin to ask these questions focus on easily-manipulated *Drosophila* species, and find no evidence of female preference for male CHCs changing across a limited range of diets [63], and temperatures [28]. However, further research will be needed here before the interplay between CHC signals and preference across environments is understood.

## 3. The Role of the Social Environment in CHC Expression

The influence of the social environment on CHC profiles is relatively poorly understood. Considering that social environment encompasses mating interactions and mating competition between conspecifics, and the clear importance of CHC profile in sexual communication, further research in this area will be important.

However, a few studies, largely focusing on *Drosophila* species, have explored CHC expression with variation in the social environment. For example, Petfield *et al.* [64] found that male *D. serrata* altered their CHC profiles within minutes of exposure to females, and experimental controls suggested that this was a male-led change, as opposed to a result of passive transfer of CHCs during physical contact and mating. Consistent with this, Gershman *et al.* [65] used a range of social environments (with presence and absence of both conspecific males and females) and found not only that male *D. serrata* CHC attractiveness varied across environments, but also that the effect of social environment differed throughout a daily cycle, showing a circadian rhythm of CHC attractiveness. It is unclear to what extent these changes in CHC attractiveness can be attributed to changes in CHC signals *versus* changes in CHC preference, but both a circadian rhythm in CHC expression and sensitivity to the social environment have been found previously in the related *D. melanogaster* [66,67]. Interestingly, there is some evidence for genetic variation for this CHC response to the social environment [66], as well as an empirical demonstration in *D. serrata* that this response can evolve [68], again suggesting that there is significant genetic variation for this plasticity.

Together, these studies clearly oppose the idea of insect CHCs as invariant and static traits. Not only do we find plasticity across abiotic environments, but we see significant changes in CHC expression or CHC attractiveness within extremely short timeframes and in response to transient changes in the social environment. Perhaps these findings should have been expected; first, because most insects express a daily cycle in sexual activity [69], the role of CHCs for signalling receptivity or attractiveness will mean that individuals will compete for mates most successfully when their CHC profile varies according to the circadian rhythm in behaviour. Second, because successful mate competition will require individuals to change their CHC expression in response to variation in social environment, the strength of sexual selection and the type of mate competition will vary extensively with exposure to different individuals and sex ratios.

The implications of changes in the social environment for sexual communication will be an important avenue for further research. Unlike abiotic environmental factors, the social environment is often transient and changeable, with the clear potential for strong selection on signalling traits through the social interactions that are intrinsic to sexual communication. Furthermore, the social environment, unlike abiotic factors, is usually subject to selection itself [70]. This is clearly illustrated by considering a focal individual and its surrounding competitors and potential mates. Both the focal individual and its “social environment” can send and receive sexual signals, and are under selection based on these signalling traits. The social environment is, therefore, likely to largely determine the intensity and direction of sexual selection on mating signals and responses. As with abiotic factors and sexual selection discussed above, it will also be important to examine how social environmental factors interact with other forces of selection.

## 4. Conclusions

The dynamic nature of insect CHC expression allows adaptive responses to changes in the environment, and this is likely to have consequences for selection on CHCs as sexual signals, as well as signal reliability across environments. Moreover, CHC plasticity enables insects to respond to the social environment, with direct implications for sexual communication, as potential mates and mating competitors largely form the social environment. In order to fully understand the role of CHCs as sexual signals, further research needs to consider CHC signalling across different social and abiotic environments, with focus on both the signaller and the receiver.
